# Evaluating Therapeutic Equivalence of Generic and Original Levetiracetam in Patients with Epilepsy: A Retrospective Study

**DOI:** 10.3390/neurolint14010022

**Published:** 2022-03-15

**Authors:** Jannapas Tharavichitkun, Tinonkorn Yadee, Poomchai Angkaow, Thanarat Suansanae

**Affiliations:** 1 Department of Pharmacy, Faculty of Pharmacy, Mahidol University, Bangkok 10400, Thailand; jannapas.cha@mahidol.ac.th; 2 Department of Pharmaceutical Care, Faculty of Pharmacy, Chiang Mai University, Chiang Mai 50200, Thailand; 3 Department of Neurology, Neurological Institute of Thailand, Bangkok 10400, Thailand; dontoro9@gmail.com; 4 Department of Pharmacy, Neurological Institute of Thailand, Bangkok 10400, Thailand; angkaewpj@gmail.com

**Keywords:** epilepsy, seizure, therapeutic equivalence, generic substitution, levetiracetam

## Abstract

The brand interchangeability of antiepileptic drugs (AEDs) is a topic of debate, especially regarding their therapeutic equivalence. This study evaluates the efficacy and tolerability of generic levetiracetam compared to the brand-name equivalent in a routine clinical setting. We conducted a retrospective study, examining patients with stable seizure frequency who received generic levetiracetam after the brand-name drug. During the six-month substitution period, changes in seizure frequency, hospitalization due to seizure exacerbation, adverse events, composite outcomes related to adjusting the AED dosage, and switching back to original levetiracetam were analyzed. Seventy-five patients were enrolled; the majority (85.3%) had focal onset seizures, and almost half (49.3%) had refractory epilepsy. Six months after the substitution, the mean seizure frequency per month was not significantly different (3.15 ± 14.47 vs. 2.77 ± 11.41; *p* = 0.970). In patients with controlled seizures before the change, the seizure frequency increased significantly (0.56 ± 1.83 vs. 0.03 ± 0.16; *p* = 0.012). Adverse events occurred in six patients. We have observed recurrent seizures or adverse events from 14 days after the transition. The original drug return rates due to recurrent seizures and adverse events were 5.3% and 1.3%, respectively. Generic levetiracetam might not show therapeutic equivalence to the original molecule, especially in patients adequately controlled by the brand-name drug.

## 1. Introduction

Levetiracetam is a second-generation antiepileptic drug (AED), widely recommended for several types of epilepsy [1]. It is rapidly absorbed with an oral bioavailability of 100%. The major metabolic pathway is enzymatic hydrolysis, which is not dependent on any liver cytochrome P450 isoenzymes. Levetiracetam is eliminated by renal excretion as unchanged, which represents 66% of the administered dose. The potential drug-drug interaction is less likely, but the findings of Perucca et al. showed that the half-life of levetiracetam is shorter in patients receiving enzyme-inducing AEDs than in those not receiving such AEDs [2,3].

Since 2018, the generic form of this AED has been introduced as an alternative to the original drug at substantially lower prices [4]. An alternative drug should feature similar biological and therapeutic equivalence, as well as pharmaceutical properties to the original [5]. Therefore, in vivo bioequivalence testing is required to confirm that the relative absorption (area under the drug concentration-time curve, AUC) and maximum plasma concentration (C_max_) are comparable to the original drug to meet regulatory approval [6].

Since August 2000, the US FDA guidelines of the biopharmaceutics classification system (BCS) suggest waiving in vivo bioavailability and bioequivalence studies for immediate-release solid oral dosage forms of generics that belong to the BCS class I (high solubility, high permeability) [7]. Levetiracetam is categorized as BCS class I; therefore, generic levetiracetam can be approved by biowaivers criteria [8].

Previous data regarding the therapeutic equivalence of generic AEDs, including levetiracetam, have shown no significant differences in seizure frequency [9,10,11,12]. Unfortunately, more recent evidence has been observed in switchback rate due to increased seizure frequency and when substituting the original levetiracetam with the generic drug in patients with epilepsy [10,11,13,14,15]. The issue of how to transition from original to generic levetiracetam in equivalent dosage has been neglected. There has also been little discussion on generic substitution in patient subgroups by their treatment responsiveness. Additionally, several neurological organizations, such as the American Epilepsy Society (Chicago, IL, USA), the Italian League Against Epilepsy (ILAE, Milan, Italy), have raised concerns about the interchangeability of each AED from various manufacturers in epileptic patients [16,17,18,19,20]. Moreover, previous studies conducted by pharmaceutical companies producing generic levetiracetam could not be applied to other manufacturers due to variations in excipients and manufacturing processes in different countries.

The first generic levetiracetam has been available in Thailand since 2015. The costs saved were estimated to be 24 THB (0.66 EUR) per 500 mg tablet by generic levetiracetam substitution. According to the hospital policy, a compulsory transition from original to generic levetiracetam was applied to all patients, regardless of their health insurance. This generic levetiracetam produced in Thailand has not been tested in previous clinical studies. Therefore, we evaluated the therapeutic equivalence of generic levetiracetam in patients with epilepsy who transitioned from the original molecule.

## 2. Materials and Methods

### 2.1. Study Design

This study followed a retrospective pre-post observational design, examining patients with epilepsy from the outpatient department of the Neurological Institute of Thailand in Bangkok, Thailand. Between February 2016 and May 2018, we screened all patients who had their medication changed from the original levetiracetam (Keppra^®^, UCB Pharma S.A., Braine-l’Alleud, Belgium) to the equivalent generic drug (Lecetam^®^, Unison Laboratories Co., Ltd., Chachoengsao, Thailand) due to hospital policy. The study protocol was approved by the institutional review boards of Neurological Institute of Thailand (protocol code 61029, 30 August 2018) and Faculty of Dentistry/Faculty of Pharmacy, Mahidol University (protocol code 2018/064.3010, 30 October 2018).

### 2.2. Study Population

Patients were eligible if they were diagnosed with epilepsy at least six months before the transition, had stable seizure frequency (within ±25% of their mean seizure frequency [21]), and had been taking a stable dose of the original levetiracetam to maintain a steady-state concentration during the 72 h before the transition. In addition, the patients have abruptly received a generic levetiracetam dose equivalent to that of the brand-name levetiracetam at the transition visit. The dosing regimen of concomitant AEDs was also not modified within 72 h before and after the substitution. Exclusion criteria were an absence seizure [22], therapy with levetiracetam formulations other than tablets before the substitution, a record of poor compliance or >80% missed doses in the medical chart, death, or loss to follow-up.

### 2.3. Outcomes

The primary outcome was the average seizure frequency per month at baseline and the 3- and 6-month follow-up visits. The secondary outcomes were recorded six months after the transition; these included the incidence of hospitalization due to recurrent seizures or adverse events and the occurrence of one or more of the following situations: adjusting the dose of generic levetiracetam, increasing the dose of concomitant AEDs, adding another AED, or returning to the original levetiracetam due to uncontrolled seizures or adverse events.

A subgroup analysis was performed to compare the primary outcome. The study population was divided into controlled and uncontrolled seizure group based on the change in seizure frequency for a minimum of three months before the transitioning visit. The controlled seizure group included patients who were seizure-free or had a ≥50% reduction in seizure frequency; the uncontrolled seizure group patients had a <50% reduction or an increase in seizure frequency [23]. Furthermore, all patients were divided into three subgroups to indicate their response to treatment based on the change in seizure frequency for a minimum of three months before the transitioning visit: positive response (decrease in frequency), stable (no change), and negative response (increase in frequency).

### 2.4. Data Collection

The patient demographic data, clinical and medical outcomes, seizure frequency, and medication adherence were extracted from medical charts and electronic databases. The terminology of seizure types, etiology, and refractory epilepsy was based on the ILAE definitions [24,25]. The pharmaceutical analysis of generic and original levetiracetam tablets was recorded from the Certificate of Analysis (COA).

### 2.5. Statistical Analysis

Based on the study by Whitehead et al. [26], a sample size of 75 patients was required to achieve 90% power at a two-sided alpha of 5% significance to detect a standardized effect size of 0.1. All analyses were based on the intent-to-treat (ITT) population. The average seizure frequency at three and six months was analyzed using the Wilcoxon signed-rank test for the primary outcome. The differences between the three treatment response groups were evaluated using the Stuart-Maxwell chi-squared test. The categorical variables were compared between the controlled and uncontrolled seizure groups using the chi-square test, whereas continuous variables with the independent *t*-test. The Kolmogorov-Smirnov test was used to determine data normality. Continuous variables of non-parametric data were analyzed using the Mann-Whitney U test in different subgroups. All data were analyzed using SPSS 18.0 (Windows version, IBM, Armonk, NY, USA).

## 3. Results

A total of 150 patients were screened, and 75 patients were included in the analysis. The mean age of all patients was 40.3 ± 17.9 years, ranging from 9 to 84 years, and 53.3% were female. The mean age at epilepsy onset was 24.4 ± 18.8 years. The majority of patients had normal renal function (creatinine clearance > 80 mL/min: 70.7%). Focal onset seizures were present in 85.3% of patients, and refractory epilepsy in 49.3%. At baseline, the mean seizure frequency per month was 2.8 ± 11.4. Fifty patients (66.7%) were included in the controlled seizure group.

In the controlled and uncontrolled seizure subgroups, the baseline characteristics were similar. Unexpectedly, the mean age was higher in the controlled seizure group (43.5 ± 18.9 vs. 33.9 ± 14.1; *p* = 0.027), and epilepsy onset was later than in the uncontrolled group (28.7 ± 20.1 and 15.7 ± 12.1, respectively; *p* = 0.001). The number of patients who had epilepsy due to symptomatic causes in the controlled seizure group was significantly higher than in the uncontrolled seizure group (84.0% and 60.0%; *p* = 0.022). The number of patients with refractory epilepsy in the controlled seizure group was significantly lower than in the uncontrolled group (32.0% and 84.0%, respectively; *p* *=* 0.000). None of the patients in the controlled seizure group underwent resective surgery after transition which was significantly less than in the uncontrolled seizure group, where 4 patients underwent surgery (0% and 16.0%; *p* = 0.005) These findings are summarized in Table 1.

Among all patients, 74.7% had a stable seizure frequency. All patients received an average dose of original levetiracetam of 1500 mg/day for 48.2 ± 33.8 months, and 77.3% also received other concomitant AEDs. Sodium channel-blocking AEDs were the agents most frequently combined in our study (50.7%; Table 2). Additionally, the median dose of the original levetiracetam in the controlled seizure group was lower than in the uncontrolled seizure group (1000 and 2000 mg/day, respectively; *p* = 0.006). Similarly, the controlled seizure group received fewer additional AEDs for polytherapy than the uncontrolled seizure group (*p* < 0.05). According to the COA of the products used in this study, the amount of levetiracetam in the brand-name drug ranges between 246.8–251.7 mg and 497.0–506.0 mg for the 250 mg and 500 mg tablets, respectively. In contrast, the generic brand had an active molecule range of 246.3–255.3 mg and 497.5–511.5 mg for the 250 mg and 500 mg tablets, respectively.

As compared to the baseline, the mean seizure frequency per month was similar at three and six months after the substitution (3.24 ± 14.49 and 3.15 ± 14.47, respectively). In addition, there was no significant difference in the mean seizure frequency after the drug transition (*p >* 0.05; Table 3). However, the mean seizure frequency in the controlled seizure group significantly increased after six months compared to the baseline (0.56 ± 1.83 and 0.03 ± 0.16, respectively; *p* = 0.012). In contrast, there was no change in seizure frequency at three and six months in the uncontrolled group compared to the baseline (8.88 ± 24.35, 8.34 ± 24.44, and 8.26 ± 18.81, respectively). There were no significant differences after six months, compared to baseline, in the median dose of levetiracetam and the number of additional AEDs in both groups. In the uncontrolled seizure group, seven patients underwent resective surgery for epilepsy. Their seizure frequency subsequently decreased, though the difference from patients with pharmacotherapy treatment alone was not significant (10.1 ± 13.7 at baseline, 4.6 ± 11.2 at three months, and 4.5 ± 11.3 at six months).

**Figure 1:**
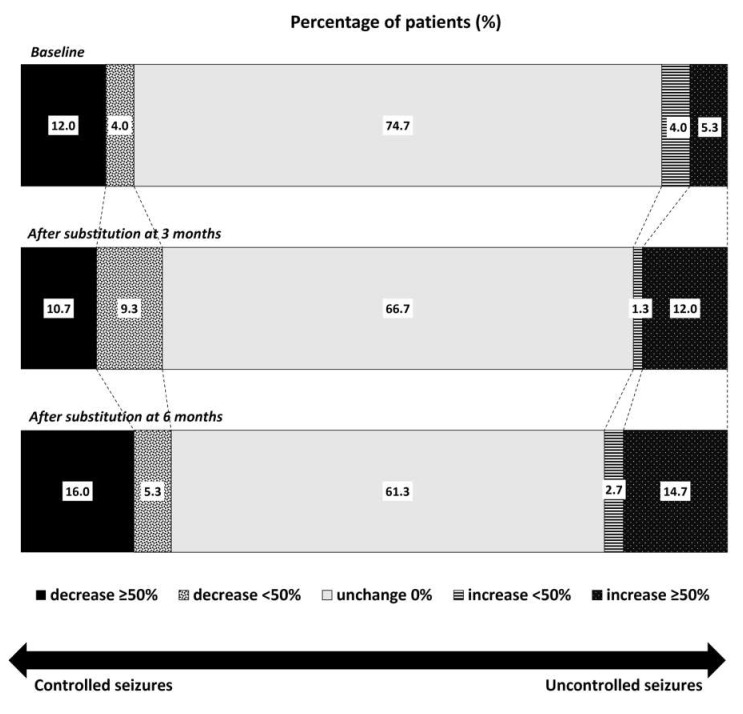
Comparison of the percentage of patients with controlled or uncontrolled seizures according to the changes in seizure frequency between baseline and three months and six months after levetiracetam substitution with the generic drug.

The number of patients with stable seizures gradually declined three and six months after the substitution (66.7% and 61.3%, respectively; Figure 1). The number of patients with a decrease in seizure frequency > 50% slightly increased, from 12.0% at baseline to 10.7% and 16.0% three and six months after the substitution, respectively. Conversely, the number of patients with ≥50% higher seizure frequency increased from 5.3% at baseline to 12.0% and 14.7% three and six months after the substitution. However, these differences were not statistically significant.

**Figure 2:**
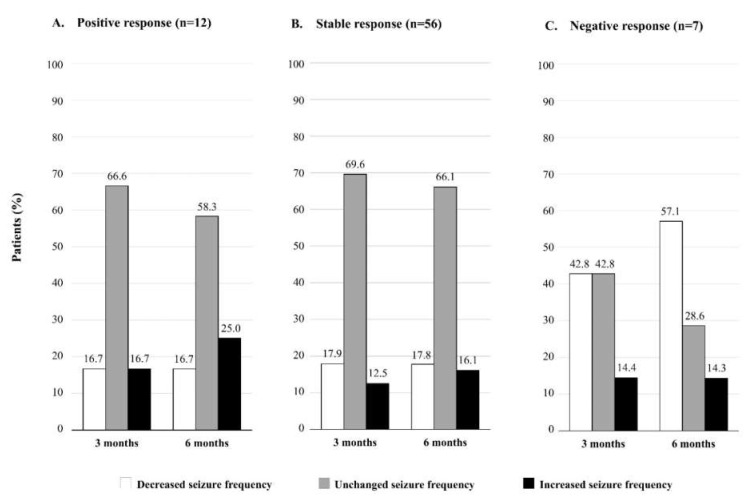
Changes in seizure frequency at three and six months after the levetiracetam substitution with the generic drug in groups based on the initial response to treatment (positive, stable, and negative).

The changes in seizure frequency at three and six months after the drug substitution are shown in Figure 2. In the group of patients with baseline positive response (*n* = 12) and stable response (*n* = 56), we found that the number of patients with higher seizure frequency increased between three and six months (from 16.7% to 25.0% in the positive response group, and from 12.5% to 16.1% in the stable response group; Figure 2A,B). On the other hand, in the group with a baseline negative response (*n* = 7), the number of patients with reduced seizure frequency increased remarkably from three to six months (from 42.8% to 57.1%; Figure 2C).

As for the secondary outcomes, six patients (8.0%) experienced adverse events in the six months after the substitution, including aggression (*n* = 2), somnolence (*n* = 1), dizziness (*n* = 2), and loss of appetite (*n* = 1). One patient was hospitalized due to recurrent seizures. Changes in therapy were necessary in 22 cases, and 54.5% were due to increased seizure frequency. Five patients returned to the original levetiracetam (increase in seizure frequency in four patients and severe dizziness in one). The earliest episode of recurrent seizure or adverse events occurred 14 days after the generic substitution.

## 4. Discussion

This retrospective study was conducted because the original levetiracetam tablet was automatically substituted with the generic equivalent following the institution’s policy established in February 2016. Only one pharmaceutical company provided generic levetiracetam in Thailand during the study. The COA of both drugs demonstrated that they contained an amount of levetiracetam within the accepted criteria (90–110% of the labeled mg amount) [27]. Therefore, the generic levetiracetam theoretically exhibits pharmaceutical equivalence to the original brand.

The efficacy of the original and generic medications was confirmed in our study. All patients presented adequate compliance and received a stable dosage regimen of all AEDs in the 72 h before and after the transition. Due to the short elimination half-life of levetiracetam (6–8 h), the steady-state concentration is achieved within 48 h [28]. Therefore, this interval was sufficient for the generic levetiracetam to reach its steady-state concentration and provide a full therapeutic response equivalent to the previous treatment.

After the transition to the generic drug, the mean seizure frequency at three and six months did not change significantly. Our result was consistent to previous studies reporting no difference in seizure frequency before and after the substitution among patients with epilepsy [9,10,11,12]. However, in our study, 13 patients (approximately 17%) suffered an increase in seizure frequency in the six months after the change.

A bioequivalence study showed similar AUC and maximum concentration (C_max_) values for the original and generic levetiracetam tablets [8]. Unfortunately, our study could not exclude an alteration of levetiracetam serum concentration due to this substitution. The conditions that can influence the AED plasma concentration are the dosing regimen, drug interactions, and changes in hepatic and/or renal function. The dosing regimen was stable in our study since the median dosage remained constant compared to baseline; other AEDs were also consistent during the study period. Moreover, levetiracetam is unlikely to have pharmacokinetic interactions with other medications because 34% of the dose is metabolized primarily in the blood by hydrolysis, and 66% is eliminated renally without molecular changes [28,29,30]. Numerous studies indicate that the concomitant use of enzyme inducers and inhibitors has no significant impact on the levetiracetam concentration; however, these interactions are clinically significant for some patients with poor seizure control which require less peak-trough fluctuation [1,2,29]. The probability of drug interactions with levetiracetam is low unless combined with enzyme-inducing AEDs [2]. In our findings, 22.7% of patients received enzyme-inducing AEDs with a constant dose throughout the study period. Therefore, drug interactions may have affected the results. Another concern is the changes in renal function. Worsening renal function leads to increased levetiracetam concentration, which may cause adverse events. The levetiracetam dosage must be individualized based on the patient’s creatinine clearance (CrCl) [28,29,30]. Most patients in our study had normal renal function (CrCl > 80 mL/min), and dosing adjustment was unnecessary. Since the median dose of generic levetiracetam and renal function were stable in our patients, the dosing unlikely affected the clinical outcomes.

In addition, variations in plasma concentrations due to variable overall chemical compositions in different brands should be considered. The generic levetiracetam approval was based on bioequivalence data determined by in vitro dissolution time profiles compared to the original brand. The latest publication by Odi et al. [31] indicates that the risk of non-bioequivalence between individual generic brands of levetiracetam available in Europe is minimal, and varying among these products is unlikely to result in clinically relevant changes in plasma drug concentrations. However, the generic brands of levetiracetam mentioned in the European study are different from the brand examined in this study. The European equivalent drugs might differ for some pharmaceutical properties, such as variable excipients and tablet hardness that impact the dissolution process. Additionally, levetiracetam absorption and oral bioavailability might have been altered if the intestinal surface area or gastric emptying time were modified in relation to seizures [32]. Therefore, the time to maximum concentration (T_max_), which represents the absorption rate and onset of action, may be altered, while the overall concentration (represented by the AUC) would remain stable [33]. Thus, the inhibition of gastric motility and esophageal peristalsis due to seizures and autonomic system alterations could affect the T_max_ of levetiracetam [34,35]. These changes may cause fluctuations in the serum levetiracetam concentration and delay the onset of action, resulting in a breakthrough seizure at the dose-ending time [36].

Regarding the steady-state plasma concentration, the prospective study by Reimer et al. [37] demonstrated non-significant variations between the original and the generic drug. The serum concentration per dose among subjects with generic levetiracetam varied between 77–140% of the original levetiracetam. Levetiracetam exhibits predictable dose linearity within the therapeutic range of 12–46 mg/L (70–270 μmol/L) from a daily dosage of 1000–3000 mg/day [38,39]. According to the pharmacokinetic model, the levetiracetam plasma concentration from the immediate-release tablet could range between 10–36 mg/L in patients with normal renal function and concurrent AED therapy, as the majority of our patients [37,40]. Therefore, the plasma concentration of generic levetiracetam could vary between 7 and 51 mg/L from baseline plasma concentrations of 10 and 36 mg/L, respectively [39]. The variable plasma concentration could cause a breakthrough seizure or side effects after the drug transition, especially in sensitive patients. In our study, 8% of patients reported adverse events, such as aggression, somnolence, dizziness, and appetite loss. These effects were dose-dependent and might be related to GABA transmission alteration, resulting from levetiracetam binding to the GABA synaptic vesicle protein 2A [41]. However, this possible pharmacokinetic aspect is a hypothesis that should be investigated based on the plasma concentration at the time of recurrent seizures or side effects.

Our findings confirm the recommendations from international guidelines (ILAE and the French Chapter of the International League Against Epilepsy [LFCE]) that changing to generic AEDs should be cautioned in seizure-free or controlled patients [17,19]. On the other hand, in almost half of the patients with a previous negative response to levetiracetam, the seizure frequency decreased at three and six months after changing to the generic drug. Among these, two patients underwent resective surgery, and the others had an increased dose of concomitant AEDs. In contrast, one-third of patients who had a baseline positive or stable response from the original levetiracetam experienced worsening seizure frequency at three and six months. Based on our results and the guidelines, there should be caution in the transition from original to generic levetiracetam in already seizure-free or controlled seizure patients.

Age and sex are critical factors to consider in epilepsy patients. Epilepsy has a bimodal distribution of age with peaks in children and the elderly. Our findings demonstrated that age and the age of epilepsy onset among patients in the controlled seizure group were significantly higher than in the uncontrolled seizure group (*p* = 0.027 and *p* = 0.001, respectively). Regarding the clinical outcomes of compulsory generic substitution to the original levetiracetam, Chaluvadi et al. reported that age in patients with epilepsy was significantly associated with switchback (increased seizure frequency or adverse events) when sex, seizure type, and treatment characteristics were controlled (*p* < 0.05) [15]. Advancing age is a predisposing factor for seizures and epilepsy due to the age-related and aging-related epileptogenic condition [42]. The elderly has an increased risk of seizures because of polypharmacy and multiple comorbidities. Moreover, women with epilepsy seem to be more sensitive to seizures because hormonal fluctuations can affect neuronal excitability [43]. These highlight the importance of considering the age group and female sex when transitioning from original to generic levetiracetam.

We suggest close monitoring for recurrent seizures and side effects starting within 14 days from the substitution. Our result of the time to events found that patients had recurrent seizures or adverse events after 14 days of transition. This time factor may be correlated with the variation in plasma concentration due to the new drug not reaching a steady-state concentration which exhibits the peak-to-trough fluctuation of plasma concentration. The study by Vari et al. [11] also reported that emerging side effects were observed within seven days from the substitution. Nevertheless, another prospective study by Reimers et al. [37] corroborated our findings. These observations confirm that the variations in levetiracetam concentration occurred within this 14-day period.

In addition to varying levetiracetam plasma concentration, psychological factors should be recognized, including placebo or nocebo effects [40]. The prospective study by Gha-Hyun et al. [10] showed that the subjects informed about the transition to generic levetiracetam might report any changes more frequently because of this awareness.

This study has some limitations. We lacked objective data, such as serum levetiracetam concentration, electroencephalography, or polysomnographic parameters, to ascertain the occurrence of seizures or adverse events [44]. The occurrence of recurrent seizures or adverse effects should be validated by standard measurement in further research. Other possible causes of increased seizure frequency, such as sleep deprivation, stress, and hormonal changes, were absent from the medical charts and could not be analyzed. Other uncontrolled confounding factors possibly affecting outcomes could be related to disease severity, comorbidities, or AED regimens. Future studies should control these factors and target epilepsy patients who did not undergo epilepsy surgery. Moreover, the number of subjects was smaller than in other studies. However, we have attempted to recruit the largest possible number of patients since the generic levetiracetam was established in the country. Our results will serve as a basic for future research with larger sample sizes. Finally, the extrapolation of our results to other levetiracetam generics or different AEDs should be performed with caution.

## 5. Conclusions

The substitution of the original levetiracetam with a generic equivalent generally resulted in similar seizure frequency after three and six months from baseline in patients receiving a stable dosage of this and other AEDs. This finding provides further evidence, that patients with no or well-controlled seizures, in particular, should be cautious when transitioning to the generic drug. Our findings suggest healthcare professionals and epileptic patients should closely monitor seizures and adverse effects, especially within 14 days after the substitution.

## Figures and Tables

**Table 1:** Baseline characteristics of total patients and patient subgroups.

Characteristics	Total Patients (*n* = 75)	Subgroup Patients		
		Controlled Seizures (*n* = 50)	Uncontrolled Seizures (*n* = 25)	*p* Value
Female, *n* (%)	40 (53.3)	26 (52.0)	14 (56.0)	0.743
Age (years), mean ± SD	40.3 ± 17.9	43.5 ± 18.9	33.9 ± 14.1	0.027 *
Weight (kg), mean ± SD	59.4 ± 17.0	59.9 ± 17.2	58.4 ± 16.9	0.722
Non-obese (BMI < 24.9 kg/m^2^), *n* (%)	54 (72.0)	36 (72.0)	18 (72.0)	1.000
Renal dysfunction (creatinine clearance) ^a^, *n* (%)				
No dysfunction (≥80 mL/min)	53 (70.7)	34 (68.0)	19 (76.0)	0.473
Mild (50–79 mL/min)	19 (25.3)	14 (28.0)	5 (20.0)	0.453
Moderate (30–49 mL/min)	2 (2.7)	1 (2.0)	1 (4.0)	1.000
Severe (<30 mL/min)	1 (1.3)	1 (2.0)	0 (0)	1.000
Age at onset of epilepsy (years), mean ± SD	24.4 ± 18.8	28.7 ± 20.1	15.7 ± 12.1	0.001 *
Type of seizure, *n* (%)				
Focal onset	64 (85.3)	43 (86.0)	21 (84.0)	0.817
Generalized onset	7 (9.3)	6 (12.0)	1 (4.0)	0.413
Unknown onset	4 (5.4)	1 (2.0)	3 (12.0)	0.105
Cause of epilepsy, *n* (%)				
Idiopathic	10 (13.3)	5 (10.0)	5 (20.0)	0.286
Symptomatic	57 (76.0)	42 (84.0)	15 (60.0)	0.022 *
Cryptogenic	8 (10.7)	3 (6.0)	5 (20.0)	0.108
Epilepsy duration ≥ 10 years, *n* (%)	46 (61.3)	27 (54.0)	19 (76.0)	0.065
Refractory epilepsy, *n* (%)	37 (49.3)	16 (32.0)	21 (84.0)	0.000 *
Epilepsy surgery, *n* (%)				
Before drug transition	5 (6.7)	2 (4.0)	3 (12.0)	0.326
After drug transition	4 (5.3)	0 (0)	4 (16.0)	0.005 *
Psychiatric disorders, *n* (%)	12 (16.0)	9 (18.0)	3 (12.0)	0.740
Anxiety	2 (2.7)	2 (4.0)	0 (0)	0.550
Depression	3 (4.0)	2 (4.0)	1 (4.0)	1.000
Hallucinations	1 (1.3)	0 (0)	1 (4.0)	0.333
Aggression	2 (2.7)	2 (4.0)	0 (0)	0.550
Irritability	1 (1.3)	1 (2.0)	0 (0)	1.000
Miscellaneous ^b^	3 (4.0)	2 (4.0)	1 (4.0)	1.000

Note: * Significant difference between subgroups (*p* < 0.05); ^a^ Classification for levetiracetam dose adjustment in patients with renal impairment; ^b^ Autism, impulsive control disorder, bipolar disorder.

**Table 2:** Antiepileptic drug treatment of total patients and patient subgroups.

Characteristics	Total Patients (*n* = 75)	Subgroup Patients		
		Controlled Seizures (*n* = 50)	Uncontrolled Seizures (*n* = 25)	*p* Value
AED use at the baseline				
Original levetiracetam dose (mg), median (Q1, Q3)	1500 (1000, 2500)	1000 (1000, 2000)	2000 (1500, 3000)	0.006 *
Polytherapy, *n* (%)	58 (77.3)	33 (66.0)	25 (100.0)	0.001 *
Concurrent AEDs (*n*), median (Q1, Q3)	1.0 (1.0, 2.0)	1.0 (0, 2.0)	2.0 (2.0, 3.0)	0.000 *
Concurrent AEDs by mode of action, *n* (%)				
Sodium-channel blocking	38 (50.7)	19 (38.0)	19 (76.0)	0.002 *
Calcium-channel blocking	4 (5.3)	2 (4.0)	2 (8.0)	0.597
GABA-receptor modulating	21 (28.0)	9 (18.0)	12 (48.0)	0.006 *
Multiple (valproate, topiramate)	32 (42.7)	16 (32.0)	16 (64.0)	0.008 *
Duration of original levetiracetam (months)				
Mean ± SD	48.2 ± 33.8	51.6 ± 33.6	41.2 ± 33.7	0.210
Range	1–127.8	2–127.8	1–108.3	
AED use at six months after substitution				
Generic levetiracetam dose (mg), median (Q1, Q3)	1500 (1000, 2500)	1000 (1000, 2500)	2000 (1000, 2500)	0.113
Polytherapy, *n* (%)	54 (72.0)	30 (60.0)	24 (96.0)	0.001 *
Concurrent AEDs (*n*), median (Q1, Q3)	1.0 (0, 2.0)	1.0 (0, 2.0)	2.0 (2.0, 3.0)	0.000 *
Concurrent AEDs by mode of action, *n* (%)				
Sodium-channel blocking	39 (52.0)	19 (38.0)	20 (80.0)	0.001 *
Calcium-channel blocking	2 (2.7)	2 (2.0)	2 (4.0)	1.000
GABA-receptor modulating	22 (29.3)	10 (20.0)	12 (48.0)	0.012 *
Multiple (valproate, topiramate)	33 (44.0)	15 (30.0)	18 (72.0)	0.001 *

Note: * Significant difference between subgroups (*p* < 0.05). AED: antiepileptic drugs.

**Table 3:** Clinical characteristics of total patients and patient subgroups.

Time Point/Variable	Total Patients (*n* = 75)	Subgroup Patients	
		Controlled Seizures (*n* = 50)	Uncontrolled Seizures (*n* = 25)
Baseline			
Seizure frequency per month			
Mean ± SD (range)	2.77 ± 11.41 (0–90)	0.03 ± 0.16 (0–1)	8.26 ± 18.81 (0.5–90)
Median (Q1, Q3)	0 (0, 1)	0 (0, 0)	2 (1, 4)
After three months			
Seizure frequency per month			
Mean ± SD (range)	3.24 ± 14.49 (0–120)	0.42 ± 1.56 (0–8)	8.88 ± 24.35 (0–120)
Median (Q1, Q3)	0 (0, 1)	0 (0, 0)	1 (0.25, 8)
*p* value (difference from baseline)	0.806	0.063	0.443
After six months			
Seizure frequency per month,			
Mean ± SD (range)	3.15 ± 14.47 (0–120)	0.56 ± 1.83 (0–10)	8.34 ± 24.44 (0–120)
Median (Q1, Q3)	0 (0, 1)	0 (0, 0)	1 (0, 3.5)
*p* value (difference from baseline)	0.970	0.012 *	0.173

Note: * Significant difference from baseline (*p* < 0.05).

## Data Availability

Not applicable.
